# Editorial: Tumorigenesis Regulated by miRNAs

**DOI:** 10.3389/fmolb.2022.933272

**Published:** 2022-06-08

**Authors:** Wei Ye, Taomei Liu, Guangchao Li

**Affiliations:** ^1^ State Key Laboratory of Applied Microbiology Southern China, Guangdong Provincial Key Laboratory of Microbial Culture Collection and Application, Guangdong Open Laboratory of Applied Microbiology, Guangdong Institute of Microbiology, Guangdong Academy of Sciences, Guangzhou, China; ^2^ Guangzhou Bio-Gene Technology Co.,Ltd., Guangzhou, China

**Keywords:** tumorigenesis, regulation, targets, miRNA, treatment

In addition to tumor-related proteins and their coding genes, non-coding genes are tightly associated with the occurrence and development of tumors. In particular, miRNAs, a class of highly conserved non-coding single-chain molecules, can inhibit gene expression at the post-transcriptional level by incomplete-complementary pairing with the untranslated regions of multiple genes, thus resulting in an imbalance of expression in a variety of tumor related genes. Therefore, miRNAs can promote or repress the expression of oncogenes or tumor-suppressor genes. These abnormal expressions detected in almost all tumor tissues can reflect their tissue origin. Previous studies demonstrated that miRNAs participate in almost every step in the occurrence and development of tumors and plays a crucial role in tumor pathogenesis.

Consequently, this topic aims to exploit novel miRNAs that could regulate the expression of tumor related genes, thus controlling the occurrence and progress of tumor. Meanwhile, the cellular, molecular and pathogenic mechanism of tumorigenesis regulation by miRNA is also to be elucidated in this topic.

The 11 articles in this Research Topic focus on the tumor genesis regulated by mRNA, which are involved in the following sections:

1) miRNAs that could promote tumorigenesis their mechanisms
2) miRNAs that could repress tumorigenesis and their mechanisms
3) The exploitation of mRNAs that could regulate tumorigenesis by bioinformatics and the demonstration of thereof function
4) Applications of miRNAs to control the occurrence and progress of tumors, and how they may affect human health.

Most articles in the Research Topic were involved in Sections 2 and 4.

For instance, the article *The Tumorigenic Properties of EZH2 are Mediated by MiR-26a in Uveal Melanoma* proposed a potential target miR-26a-EZH_2_ axis for the development and treatment strategies for uveal melanoma (Li et al.). The results indicated the downregulation of miR-26a and upregulation of EZH_2_ in uveal melanoma. Moreover, the overexpression of miR-26a and the knockout of EZH_2_ can suppress the proliferation for uveal melanoma cells, meanwhile, the knockout of EZH_2_ can counteract the tumor inhibition effect *via* the overexpression of miR-26a, suggesting that EZH_2_ is the direct target for miR-26a. Furthermore, EZH_2_ interacting proteins (UBC, CDK1, HDAC1, SUZ12, EED) were also found to participate in miR-26a-mediated tumor progression. However, the molecular mechanism of miR-26a-EZH_2_ still needs further investigation by *in vivo* experiments.

In 2020, there were 910,000 cases of liver cancer worldwide, which was the sixth most common malignant tumor in the world. Liver cancer kills 830,000 people, making it the third leading cause of cancer deaths. Thus it is stringent to develop novel strategies and targets for hepatocellular carcinoma. The manuscript *Delivery of miR-26a Using an Exosomes-Based Nanosystem Inhibited Proliferation of Hepatocellular Carcinoma* offers a new exosome delivery system based miRNA antitumor therapy strategy (Mahati et al.). In this article, the appearance of drug-carrying exosomes were observed by electron microscopy and dynamic light scattering. Moreover, anti-GPC3 scFv-modified exosomes can effectively and selectively delivered miR-26a to GPC3-positive hepatocellular carcinoma cells, thus inhibiting the cell migration and proliferation by the regulation of miR-26a downstream genes. The *in vitro* and *in vivo* experiments confirmed the high effeciency and selectivity of exosomes for the delivery of miR-26a and *via* the modification of exosomes with tumor specific antibodies.

In addition, there is another manuscript also described another exosomes based delivery system, which was titled as *Delivery of Anti-miRNA-221 for Colorectal Carcinoma Therapy Using Modified Cord Blood Mesenchymal Stem Cells-Derived Exosomes* (Han et al.). This article described an anti-miRNA-221 oligonucleotide (AMO) loaded exosomes, which can effectively suppress the proliferation of clonal formation of colon cancer cells *in vitro*. The results of a xenograft tumor model also showed that iRGD-modified exosomes were obviously enriched in tumor sites, exerting excellent anti-tumor efficacy. *In vivo* imaging showed that exosomes were mainly distributed in liver, spleen, and lung tissues. This article suggested that genetically modified exosomes could be served as an ideal natural nanostructure for anti-miRNA oligonucleotide delivery.

Acute myeloid leukemia causes great threaten to human health, more and more young adults suffered from acute myeloid. Therefore, it is urgent to exploit novel miRNAs to reduce the hazard of acute myeloid leukemia towards human health. The manuscript *MiRNA-142-3P and FUS can be Sponged by Long Noncoding RNA DUBR to Promote Cell Proliferation in Acute Myeloid Leukemia* reported the biological functions of lncRNA DUBR in AML pathogenic mechanism (Yin et al.). The knockdown of DUBR with small interfering RNA (siRNA) led to the suppression of survival and colony formation ability, as well as induction of apoptosis, in AML cells. And the downregulation of DUBR promoted the expression of FUS protein, targeting inhibition of FUS significantly promoted cell apoptosis in AML cell lines, indicating that DUBR is the potential target for AML therapy.

Exosome can be also employed to improve the miRNA therapy efficiency for tumor. Thus, the manuscript *Exosome-Transmitted miR-128 Targets CCL18 to Inhibit the Proliferation and Metastasis of Urothelial Carcinoma* investigated the regulatory function of exosome-transmitted miR-128 and chemokine (C-C motif) ligand 18 (CCL18) on urothelial carcinomas (UCs) (Shang et al.). The exosome-transmitted miR-128 can inhibit cell proliferation, invasion, and migration in UCs, as well as the apoptosis mediated *via* BUCT24, and these effects can be reversed by CCL18. Meanwhile, miR-128 can also inhibit the proliferation (*p* < 0.05) and metastasis (*p* < 0.05) of UCs in nude mice, thus providing a new target and therapeutic strategy for UCs treatment.

Besides the miRNA therapy for different tumor, there are also some articles involved in the diagnosis of different types of tumors, which is also important for the cancer treatment. The manuscript *miRNA Combinatorics and its Role in Cell State Control-A Probabilistic Approach* proposed an overlooked quantitative dimension for a set of genes and miRNA regulation in living cells, this study disclosed that the modest miRNA overexpression resulted in the shift of cell identity and cancer evolution (Mahlab-Aviv et al.). The findings in this study revealed that most genes are resistant to miRNA regulation, whereas the composition of the abundant miRNAs dictates the state of the cells, indicating the role of miRNAs in cell state. Moreover, FAT10 was reported as a biomarker for the tumor immune infiltration in skin cutaneous melanoma (SKCM) in the manuscript *FAT10 is a Prognostic Biomarker and Correlated With Immune Infiltrates in Skin Cutaneous Melanoma* (Wang and Zhang). *FAT10* gene expression level is positively related to immune infiltration, immune checkpoint expression, whereas negatively related to tumor cell invasion and DNA damage. These results indicated *FAT10* gene as an effective biomarker for the diagnosis and treatment of SKCM.

Furthermore, there are four review summarized the role of miRNA in the cell proliferation, apoptosis, invasion, therapeutic targets, and the pathogenicity mechanism of different types of cancers in this Research Topic, which can facilitate the diagnosis and therapy of different kinds of tumor genesis and progress employing different miRNAs. The targets of these miRNAs can thus provide more effective strategies for the treatment of different cancers.

*Cervical Cancer, Papillomavirus, and miRNA Dysfunction* summarized miRNAs that affected the HPV infection process and miRNAS contributing to the development and maintenance of malignant cervical tumor cells (Bañuelos-Villegas et al.). Meanwhile, the miRNAs served as biomarker for precancerous lesions or cervical tumors were also recapitulated in this review, which can facilitate the early diagnosis and treatment of cervical cancer *via* miRNAs.

Moreover, the other three reviews were prepared by Mohammad Taheri’s. team. miR-1246 is the firstly recognized microRNA through a high throughput sequencing technique in human embryonic stem cells. *A Review on the Role of miR-1246 in the Pathoetiology of Different Cancers* focuses on the *in vivo* and *in vitro* oncogenic roles of miR-1246 in colorectal, breast, renal, oral, laryngeal, pancreatic and ovarian cancers as well as melanoma and glioma (Ghafouri-Fard et al.). The regulatory roles of miR-1245 in signal pathways of RAF/MEK/ERK, GSK3β, Wnt/β-catenin, JAK/STAT, PI3K/AKT, THBS2/MMP and NOTCH2 miR-1246 have been demonstrated. And this review can deepen our scientific understanding of miR-1246 systematically. miR-1290 is transcribed from *MIR1290* gene on the chromosome 1p36.13. *A Review on the Role of miR-1290 in Cell Proliferation, Apoptosis and Invasion* summarized *in vitro, in vivo* and human investigations on the miR-1290 in the tumorgenesis (Ghafouri-Fard et al.). Moreover, the regulatory activity of miR-1290 in JAK/STAT3, PI3K/AKT, Wnt/β-catenin and NF-κB pathways were also summed up, which can promote the application of miR-1290 in the diagnosis and treatment in tumorgenesis. Breast cancer exhibited a higher and higher incidence in the worldwide. Angiogenesis plays crucial role in the development of breast cancer, and miRNA has been reported to serve as significant role in the angiogenesis process, thus it is important to investigate the linkage of miRNA and breast cancer. *MicroRNAs: Important Players in Breast Cancer Angiogenesis and Therapeutic Targets* described the recent progress of miRNA signature and thereof targets in the development of breast tumor (Hussen et al.). Furthermore, the miRNA-base strategies for the treatment of breast cancer targeting anti-angiogenic response were also discussed in this review, which is favorable to the future novel miRNAs-based therapeutic strategies for the breast cancer.

